# RCAF-Net: Wildlife Target Detection in Complex Forest Scenarios

**DOI:** 10.3390/ani16101484

**Published:** 2026-05-12

**Authors:** Xiuling Yu, Chenxiao Qu, Yifu Xu, Senyue Guo, Lili Fu, Yang Zhou

**Affiliations:** 1 College of Information and Technology, Jilin Agricultural University, Changchun 130118, China; yuxiuling@jlau.edu.cn (X.Y.); qcx@mails.jlau.edu.cn (C.Q.); yifuxu@mails.jlau.edu.cn (Y.X.); 20251663@mails.jlau.edu.cn (S.G.); 2 College of Biological and Agricultural Engineering, Jilin University, Changchun 130022, China; full23@mails.jlu.edu.cn

**Keywords:** wildlife detection, complex forest ecosystems, small target detection, edge deployment, feature fusion

## Abstract

This study focuses on wildlife target detection in complex forest environments in Northeast China, where monitoring images are often affected by background interference, frequent occlusion, and large target scale variation. In addition, distant animals usually appear as small targets with limited local detail information, increasing the difficulty of reliable detection. To address these challenges while maintaining deployment efficiency, an improved wildlife target detection model, RCAF-Net, is proposed based on YOLO11n. The proposed method enhances shallow feature representation, strengthens multi-scale contextual modeling, improves cross-layer feature fusion consistency, and introduces a lightweight detection head to balance detection accuracy and computational cost. Experimental results on a self-built dataset of eight typical wildlife species show that RCAF-Net achieves Precision, Recall, mAP@0.5, and mAP@0.5:0.95 values of 89.3%, 78.4%, 87.3%, and 67.3%, respectively, improving upon YOLO11n by 4.1%, 2.6%, 3.9%, and 3.4%. On the Wildlife Computer Vision Model dataset, the proposed model also achieved improved generalization performance in cross-dataset testing. In addition, the model operated at approximately 27 FPS on the Jetson TX2 NX platform. These results suggest that RCAF-Net has potential applicability for automated wildlife monitoring in complex forest environments.

## Simple Summary

Wildlife monitoring is important for biodiversity conservation and ecological management. However, images collected in natural forest environments are often affected by complex backgrounds, occlusion, and distant small targets, making automatic wildlife detection difficult. To address these challenges, this study proposes RCAF-Net, an improved wildlife target-detection model based on YOLO11n for complex forest monitoring scenarios in Northeast China. Experimental results showed that the proposed method improved detection performance while maintaining relatively low computational complexity. In addition, the model demonstrated good inference efficiency on an embedded edge device, indicating its potential applicability for edge-assisted wildlife monitoring in forest ecosystems.

## 1. Introduction

Forest ecosystems play a vital role in maintaining ecological balance, protecting biodiversity, and providing ecosystem services. As key components of these ecosystems, wildlife populations, spatial distributions, and activity patterns reflect regional ecological quality and biodiversity status. The ongoing impacts of human expansion, habitat destruction, and climate change have heightened the significance of wildlife monitoring for ecological conservation and resource management [1]. Long-term, stable, and efficient wildlife monitoring aids in understanding species population dynamics and behaviors [2]. It also provides essential data to support species protection, habitat management, and the formulation of ecological conservation policies [3].

Traditional wildlife monitoring methods mainly include manual surveys, mark-recapture, and radio tracking. Although these approaches played important roles in early ecological research, they often involve high labor and time costs and may disturb normal animal activities [4,5]. With the development of automated monitoring technologies, camera traps, infrared cameras, and other imaging devices have been widely deployed, leading to the rapid accumulation of large volumes of wildlife image and video data [6]. However, images collected in natural environments are frequently affected by complex backgrounds, illumination variation, occlusion, and unstable image quality, which increases the difficulty of manual screening and automated analysis [7]. Consequently, recent advances in computer vision have provided new technical approaches for automated wildlife image analysis. Among them, object detection methods have shown considerable potential for improving monitoring efficiency and reducing manual workload in large-scale wildlife monitoring tasks [8].

Existing object detection methods can generally be categorized into two-stage and single-stage approaches according to their detection pipelines [9]. Two-stage methods, represented by Faster R-CNN, usually achieve strong candidate region modeling and object localization performance through multi-stage feature refinement [10]. Several studies have applied such methods to wildlife monitoring tasks. For example, Ma et al. [11] improved herbivore detection in low-altitude UAV imagery by incorporating HRNet and optimized anchor strategies into Faster R-CNN, while Lyu et al. [12] enhanced deer detection in thermal infrared UAV images using customized anchor boxes and multi-scale RoIAlign fusion, achieving a small-target mAP of 78.9%. Although these methods improved detection accuracy for specific scenarios, they generally rely on additional region proposal generation, overlap processing, and multi-stage feature refinement, resulting in more complex detection pipelines and longer inference time. Such characteristics reduce their efficiency and limit their applicability in resource-constrained wildlife monitoring scenarios.

In contrast, single-stage methods represented by the YOLO series integrate object localization and classification within a unified framework, offering advantages in inference speed and deployment efficiency [13]. Therefore, they have been widely adopted in wildlife monitoring tasks. Existing studies mainly focus on three aspects: small-target detection, feature enhancement under complex backgrounds, and lightweight deployment.

For small-target perception, Lei et al. [14] improved a YOLOv7-based waterbird detection model and achieved an approximately 5% increase in mAP, while Ye et al. [15] enhanced shallow feature extraction and reduced missed detections in UAV scenarios by introducing an additional P2 detection layer. To improve feature representation under complex backgrounds and occlusion conditions, Yang et al. [16] incorporated channel attention and Swin Transformer into YOLOv5s, whereas Zhu et al. [17] combined BiFPN and MHSA to improve detection stability in complex forest environments. However, these improvements also increased model complexity to different extents. For example, the detection speed reported by Yang et al. [16] decreased from 53 FPS to 40 FPS, while the model size increased from 14.6 MB to 15.2 MB. Similarly, the GFLOPs of YOLO-WildASM proposed by Zhu et al. [17] increased from 8.1 to 12.5. In addition, He et al. [18] explored lightweight deployment strategies for thermal infrared UAV wildlife detection, although the validation scenarios remained relatively limited.

Despite significant advances in wildlife target detection in field scenarios, several critical issues persist in practical monitoring applications. Environments such as forests, snowy landscapes, and bushes often present complex background textures. This is particularly challenging in monitoring tasks for specific species like the Amur tiger and Amur leopard, where the high similarity between target textures and backgrounds complicates recognition [19]. Additionally, frequent occlusions can weaken the effective response of target regions, leading to false positives and missed detections. Wildlife targets also exhibit significant scale variations, with distant small targets lacking detailed information. Inconsistent information representation during multi-scale feature fusion can adversely affect localization accuracy and detection stability [20]. Furthermore, field monitoring typically relies on resource-constrained edge devices, necessitating a balance between enhanced detection performance, parameter size, computational complexity, and real-time deployment requirements [21].

To address these challenges, this study introduces RCAF-Net (Receptive-field and Context Alignment Fusion Network), a wildlife target detection model specifically designed for complex field scenarios, using YOLO11n as the baseline architecture. The key contributions and innovations of this work are as follows:

(1) A wildlife detection dataset was established for typical complex forest regions in Northeast China. This dataset encompasses various natural environments, including forests, snowy landscapes, and bushes, and features eight representative wildlife species. It effectively reflects the challenges encountered in real-world monitoring, such as strong background interference, significant scale variations, frequent occlusions, and high similarity between targets and their environments. This dataset provides a foundational resource for model training, performance evaluation, and subsequent method improvements.
(2) An improved wildlife target detection framework based on YOLO11n was developed for complex forest monitoring scenarios. The proposed framework incorporates PMGHA, RFAConv, CSFCN, and ELGH to enhance feature representation, multi-scale feature fusion, and lightweight deployment capability under complex natural environments. By jointly optimizing detection performance and deployment efficiency, the proposed method improves the robustness and adaptability of wildlife target detection in complex forest scenes.
(3) Extensive experiments demonstrate that RCAF-Net achieves improved detection performance while maintaining good deployment efficiency in complex forest scenarios. In addition, visualization analysis and deployment validation further confirm the effectiveness and practical applicability of the proposed method for wildlife monitoring tasks.

## 2. Materials and Methods

**Figure 1:**
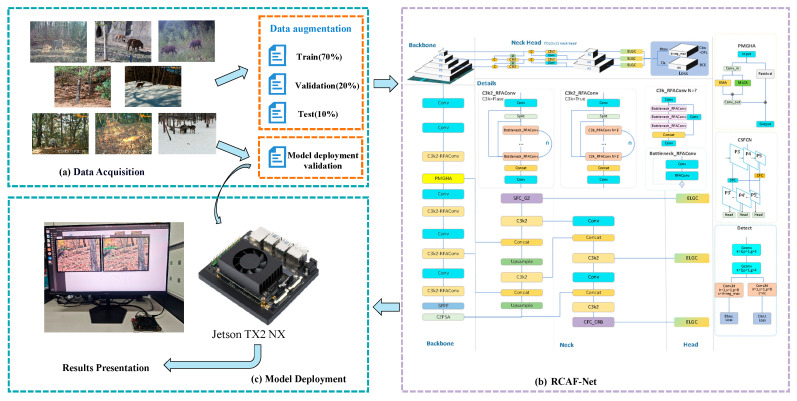
Implementation process of RCAF-Net for detecting wildlife targets in complex forest environments.

Figure 1 illustrates the implementation process of the proposed wildlife target detection method based on RCAF-Net. It encompasses three main components: dataset construction, model training and optimization, and edge deployment validation. This figure summarizes the overall technical framework from image acquisition to practical application of the model.

### 2.1. Data and Preprocessing

**Figure 2:**
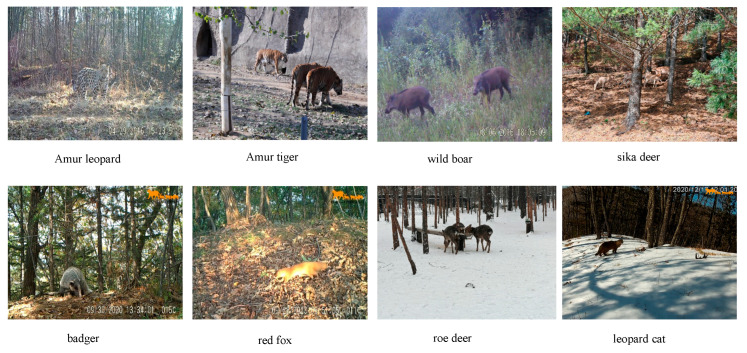
Sample images of different wildlife species.

Northeast China retains a relatively intact montane forest ecosystem, featuring diverse habitats such as forests, shrublands, river valleys, and winter snow cover. These environments provide stable conditions for various wildlife species [22]. Notably, the Amur Tiger and Leopard National Park and its surrounding areas are home to rare large carnivores, including the Amur tiger and Amur leopard, as well as significant populations of sika deer, wild boars, roe deer, red foxes, badgers, and leopard cats [23,24]. Based on the ecological importance and regional characteristics of wildlife species in Northeast China forest ecosystems, this study constructed a target detection dataset comprising eight representative wildlife species: Amur tiger, Amur leopard, sika deer, wild boar, red fox, roe deer, leopard cat, and badger. The selected categories encompass large carnivores, ungulates, and medium-sized carnivores, reflecting the typical wildlife composition and monitoring targets of forest ecosystems in Northeast China. In addition, these species exhibit differences in target scale, appearance, and spatial distribution under complex natural scenes, with some examples illustrated in Figure 2.

To construct a wildlife target detection dataset for Northeast China, images were collected from three sources. First, key frames from field monitoring videos in the Amur Tiger and Leopard National Park [25] provided authentic visual samples under real monitoring conditions. Second, documentary stills supplemented images of targets in various poses, perspectives, and behavioral states. Third, publicly available images from the internet enriched the dataset with scarce category samples and expanded the diversity of appearance features. The integration of these multi-source samples resulted in a dataset characterized by significant diversity in resolution, target scale, lighting conditions, and background textures, covering typical field scenarios such as forests, snowy landscapes, and shrublands.

Data screening and cleaning revealed differences in composition, clarity, shooting distance, and target prominence. These differences caused inevitable domain shifts across key frames, documentary stills, and public images. To mitigate the impact of this factor on experimental evaluation, samples from different sources were uniformly statistically processed during data organization. The composition of each subset was controlled during data partitioning to reduce dependency on a single source sample in the evaluation results. The test set primarily featured samples from real monitoring scenarios, with monitoring key frames, documentary stills, and internet images comprising 60%, 20%, and 20%, respectively. In the actual partitioning process, sample source composition for the test set was prioritized, followed by stratified sampling within each subset to balance source distribution and class representation. Additionally, considering the high reliability requirements of species-level target annotations, researchers from the College of Animal Science and Technology at Jilin Agricultural University participated in species identification and label verification for challenging samples. Ambiguous samples were independently reviewed and rechecked by researchers with relevant wildlife identification experience to improve annotation consistency and reduce labeling errors caused by inter-class similarity.

After screening and organization, a target-detection dataset comprising eight wildlife species was established, consisting of 4098 images. To ensure consistency in model training and evaluation, images were stratified by category into training, validation, and test sets approximately in a ratio of 7:2:1. All images were manually annotated using the LabelImg (version 1.8.6) tool and uniformly saved in YOLO format. Table 1 presents the statistics of the original image counts for the eight wildlife species across the training, validation, and test sets.

**Figure 3:**
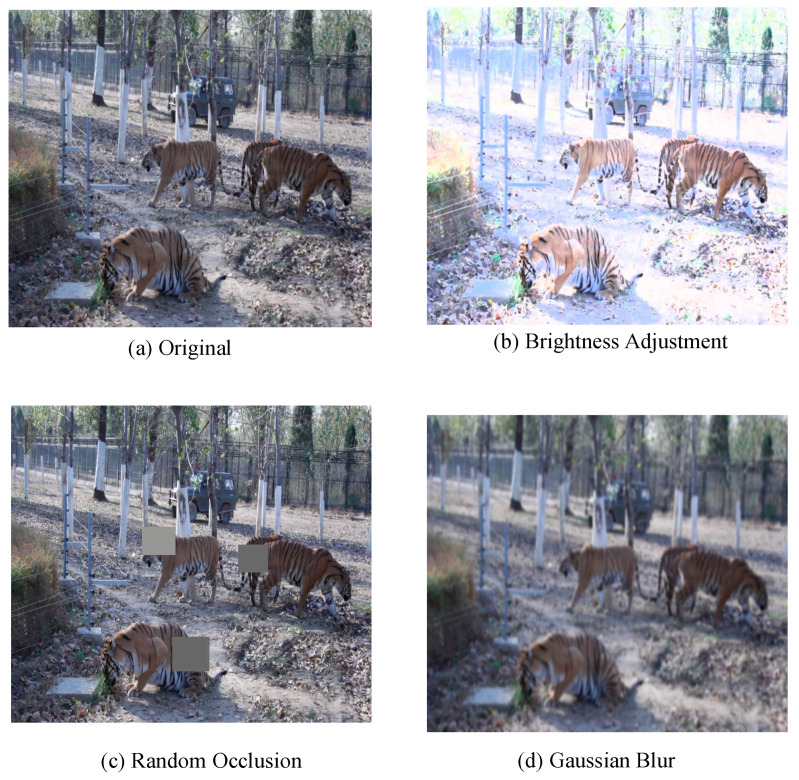
Examples of augmented training set images: (**a**) Original image; (**b**) Brightness adjustment; (**c**) Random occlusion; and (**d**) Gaussian blur. The grey boxes in (**c**) indicate the masked regions generated during the random occlusion augmentation process.

To enhance the model’s detection robustness and generalization ability in complex natural scenes, offline data augmentation was applied exclusively to the training set, while the validation and test sets retained their original data distribution for objective performance assessment. Specifically, Gaussian blurring, random occlusion, and brightness adjustment with moderate augmentation intensity were employed to simulate common disturbances in field monitoring environments, with representative augmentation examples illustrated in Figure 3. Brightness adjustment was randomly applied within approximately ±20% intensity variation, while random occlusion regions generally covered approximately 5–15% of the target area to avoid excessive distortion of target appearance. As a result, the training set size increased from 2865 images to 8595 images.

### 2.2. Construction Process of RCAF-Net

YOLO11 is a representative version of the YOLO series, featuring multiple structural optimizations while inheriting the design principles of its predecessors [26]. It retains the three-stage architecture of Backbone, Neck, and Head. In the Backbone section, YOLO11 replaces the original C2f structure with the C3K2 module, achieving a better balance between feature extraction capability and parameter scale. Following the SPPF module, the C2PSA structure is introduced, integrating the concept of Pyramid Spatial Attention (PSA) [27] to enhance responsiveness to critical areas and improve feature representation. In the Head section, the classification branch employs depthwise separable convolution (DWConv) [28] to reduce redundant computations, while the regression branch maintains conventional convolution to ensure target localization accuracy. Overall, YOLO11 achieves a favorable balance between detection performance and inference efficiency. Based on this, the lightweight version YOLO11n is chosen as the baseline model for further improvements aimed at complex forest scenarios.

**Figure 4:**
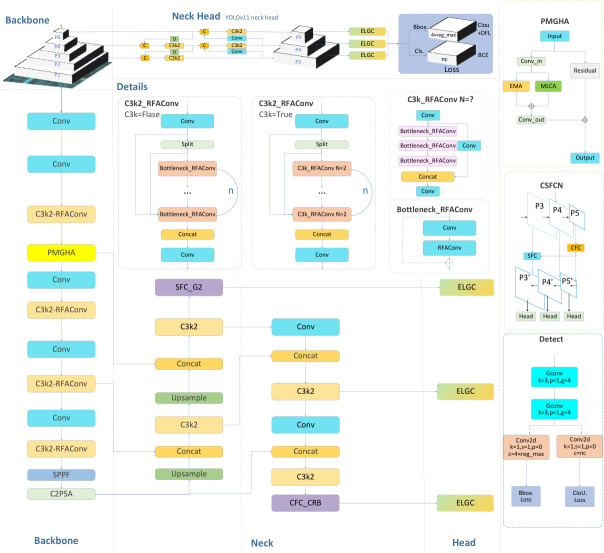
Diagram of the RCAF-Net model.

RCAF-Net is built upon YOLO11n, with the overall architecture illustrated in Figure 4. Considering the challenges of complex background interference, distant small-target perception, unstable multi-scale feature fusion, and deployment constraints in forest monitoring scenarios, the proposed method focuses on improving feature representation, enhancing feature fusion stability, and maintaining lightweight deployment efficiency. To achieve these objectives, structural optimizations are introduced in the Backbone, Neck, and Head sections of the network, corresponding to feature extraction, feature fusion, and deployment efficiency, respectively.

In the Backbone, the PMGHA module is introduced to strengthen target-related feature responses and suppress background interference, while RFAConv [29] is incorporated to improve local detail perception and contextual information modeling. In the Neck, the CSFCN (Context and Spatial Feature Calibration Network) is integrated to enhance semantic consistency and spatial coherence during multi-scale feature fusion [30]. In the Head, the ELGH detection head is constructed to maintain target localization capability and inter-channel information interaction while reducing parameter scale and computational overhead.

#### 2.2.1. C3K2-RFAConv Backbone Feature Extraction Module Based on Receptive-Field Attention

In complex natural scenes, wildlife targets often exhibit significant scale variation, frequent pose changes, and irregular contour structures. For distant small targets, local structural information, such as the edges of the head, limbs, and torso, is inherently weak. When targets experience further occlusion, deformation, or changes in perspective, conventional convolution, which uses fixed kernels for uniform modeling of local neighborhoods, struggles to adaptively adjust the importance of different positions within the receptive field based on variations in target shape and local structure. This limitation restricts the effective representation of crucial local details and multi-scale contextual information.

To address this issue, RFAConv is introduced into the C3K2 unit of the YOLO11n backbone network, forming the C3K2-RFAConv structure to replace certain convolutional mappings within the original C3K2 unit. This design maintains the existing structural organization while integrating the receptive-field attention mechanism into the feature extraction process. Consequently, the network can perform differentiated modeling of local neighborhood responses based on input content, enabling more effective representation of the irregular contours and critical local structural features of wildlife targets.

**Figure 5:**
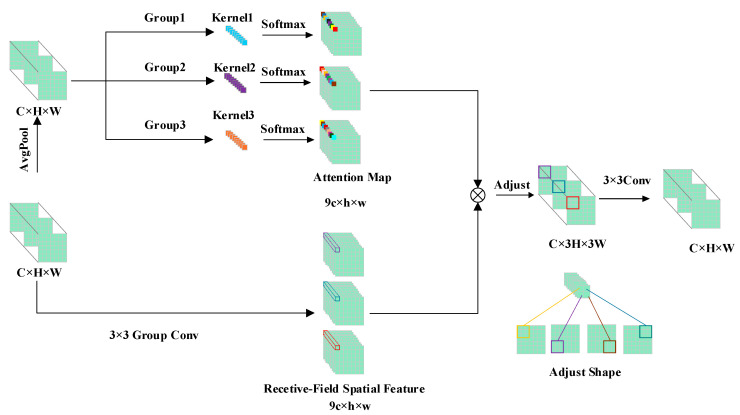
Structure diagram of RFAConv.

Figure 5 illustrates the RFAConv structure. For the input features X, the module first extracts the corresponding 3 × 3 local receptive field information around each spatial position. Each 3 × 3 convolution kernel contains 9 sampling locations, allowing the receptive field spatial features in the intermediate representation to be viewed as extending each channel into 9 position responses, represented by the dimension 9C × H × W. Subsequently, the module generates the receptive field attention map $A_{rf}$ through average pooling, 1 × 1 grouped convolution, and Softmax. Simultaneously, the receptive field spatial features $F_{rf}$ are obtained via 3 × 3 grouped convolution, batch normalization, and an activation function. Finally, the attention map $A_{rf}$ is element-wise multiplied with the receptive field spatial features $F_{rf}$ to produce the recalibrated local response features *F*. The process can be expressed as Equation (1):

(1) $$\begin{array}{c} F=A_{rf}⊙F_{rf}\\ \;\;=\mathrm{Softmax}\left(g_{1\times 1}\left(AvgPool\left(X\right)\right)\right)⊙\delta \left(BN\left(g_{3\times 3}\left(X\right)\right)\right) \end{array}$$

Here, $A_{rf}$ represents the receptive field attention map, while $F_{rf}$ denotes the receptive field spatial features. The mappings $g_{1\times 1}$ and $g_{3\times 3}$ correspond to the 1 × 1 and 3 × 3 grouped convolution operations, respectively. The function δ(·) indicates the activation function, and ⊙ signifies element-wise multiplication.

Due to the organization of the weighted receptive field responses in an unfolded format, a subsequent shape rearrangement is required to transform the dimensions from 9C × H × W to C × 3H × 3W. This adjustment maps the previously stacked local receptive field positions in the channel dimension back to the spatial dimension. Following this, a 3×3 convolution is applied for local aggregation, resulting in the final output features *Y*, as shown in Equation (2):

(2) $$Y={Conv}_{3\times 3}\left(Adjust\left(F\right)\right)$$

Here, Adjust(⋅) denotes the operation that rearranges the shape of the weighted receptive field responses to restore a spatial organization suitable for subsequent convolutional aggregation.

Unlike conventional convolution, which applies uniform weighting within the receptive field, RFAConv adapts the responses of different positions in the local neighborhood based on input content. This allows for differential emphasis on regions critical for target discrimination. In wildlife detection tasks, this module enhances the representation of local structures, such as the edges of the head, limbs, and torso, while improving the backbone network’s ability to model shape features, local details, and multi-scale contextual information.

#### 2.2.2. PMGHA Shallow Feature Enhancement Module Based on Parallel Mixed Attention

The shallow layers of the backbone network are crucial for extracting target detail information, primarily encoding low-level features such as edges, textures, and local structures. The quality of these outputs directly influences subsequent semantic modeling and multi-scale feature fusion. However, in complex forest scenes, factors such as snow glare, branch shadows, bush textures, and local occlusions can produce strong responses at the shallow layer, leading to over-activation of non-target areas, increasing the risk of false positives, and undermining effective representation of true wildlife contours and structural information. To address this issue, an Efficient Parallel Multi-Granularity Hybrid Attention (PMGHA) module is introduced in the shallow layers of the backbone network. This module combines two branches, Efficient Multi-Scale Attention (EMA) [31] and Mixed Local-Channel Attention (MLCA) [32], in parallel to jointly recalibrate input features across spatial and channel dimensions. The EMA branch focuses on spatial-feature recalibration, while the MLCA branch emphasizes channel feature selection and enhancement. Their synergistic effect helps retain target detail information while improving the discriminability of shallow features, thereby enhancing the separability between wildlife targets and complex backgrounds.

**Figure 6:**
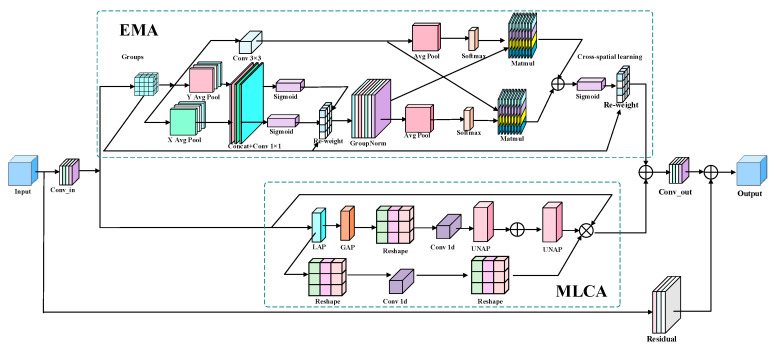
Structure diagram of PMGHA.

The overall structure of PMGHA consists of three parts: input alignment, parallel attention enhancement, and residual correction. As shown in Figure 6, let the input features be denoted as X. Initially, a 1×1 convolution is applied for channel alignment, resulting in the base feature F, as shown in Equation (3):

(3) $$F\;=\;\delta ({\mathrm{BN}(\mathrm{Conv}}_{1\times 1}(X)))$$

In the equation, δ(·) denotes the SiLU activation function. Subsequently, F is fed into both the EMA branch and the MLCA branch for spatial and channel recalibration, respectively. The outputs from both branches are summed, followed by a 1×1 convolution for integration. Finally, the result is fused with the input in a residual manner to produce the module output Y, as shown in Equation (4):

(4) $$Y\;=\;X\;+\;\delta \left(\mathrm{BN}\left({\mathrm{Conv}}_{1\times 1}\left(M(F)\;+\;E(F)\right)\right)\right)$$

Here, M(·) and E(·) represent the mapping processes of the MLCA and EMA branches, respectively.

The MLCA branch focuses on feature selection along the channel dimension. This branch simultaneously extracts local and global pooled features and employs lightweight 1D convolutions to model inter-channel correlations. After fusing the local and global channel responses, the features are mapped back to the input resolution, resulting in the final channel attention A. This process can be expressed as Equation (5):

(5) $$A\;=\;P_{H,W}\left(\alpha A_{l\;}+\;(1\;-\alpha )A_{g}\right),\;M(F)\;=\;F⊙A$$

Here, $A_{l}$ and $A_{g}$ denote the local and global channel attention, respectively, while α represents the local weight coefficient. $P_{H,W}\left(\cdot \right)$ indicates the mapping of attention to the input feature resolution. This branch enhances effective channels associated with wildlife targets while suppressing redundant channel responses that are strongly influenced by background interference.

The EMA branch is primarily designed to suppress spatial redundancy in complex backgrounds. As illustrated in Figure 6, this branch first partitions the input features into multiple groups by channel. Within each group, contextual information is extracted along the height and width dimensions to capture direction-related features. A spatial weight map is generated by combining these local convolutional features, enabling directional-aware weighted modulation of the group features. This process can be expressed as Equation (6):

(6) $$E(F)\;=\;{\mathrm{Reshape}}^{-1}(\mathrm{Reshape}(F)⊙W_{s})$$

In the equation, Reshape Reshape(F) denotes the rearrangement of input features F into group representations, while $W_{s}$ represents the spatial weight map generated by modeling directional context and local convolutions. The symbol ⊙ indicates element-wise multiplication, and ${\mathrm{Reshape}}^{-1}(\cdot )$ signifies the restoration of the weighted group features to their original arrangement. This approach enables the EMA branch to suppress abnormal activations caused by background textures and local noise during the shallow processing stage, while enhancing spatial responses related to target contours and structures.

In summary, PMGHA introduces the EMA and MLCA branches in parallel to achieve coordinated correction in both spatial and channel dimensions. This module effectively reduces interference from factors such as snow glare, branch shadows, and background textures during the shallow processing stage. It enhances the discriminability of shallow features while preserving essential target detail, thereby providing a more stable input representation for subsequent feature extraction, multi-scale fusion, and object detection.

#### 2.2.3. Context Calibration and Spatial Alignment-Based CSFCN Feature Fusion Module

In complex natural scenes, wildlife targets often exhibit significant scale differences, with small distant targets and larger nearby targets frequently coexisting. This situation imposes greater demands on multi-scale feature fusion. During the feature pyramid propagation process, higher-level features possess strong semantic expressiveness, making them suitable for representing larger or more complete targets. Conversely, lower-level features retain richer edge, texture, and positional information, which is critical for identifying small-scale targets. However, direct fusion of features from different levels can lead to semantic discrepancies and spatial misalignment between high-level semantic responses and low-level fine-grained information. This may weaken small-scale targets during the fusion process or even obscure them with stronger high-level semantic responses, compromising the stability of feature representation in complex scenes.

YOLO11 employs a PAN structure for multi-scale feature propagation, which alleviates the impact of scale differences to some extent. However, its capability for modeling contextual relationships and accurately aligning cross-layer features remains limited in complex natural scenes. To address these issues, this paper introduces the Context and Spatial Feature Calibration Network (CSFCN) into the PAN pathway. By performing contextual semantic calibration and spatial alignment on multi-layer features, CSFCN enhances the correspondence between high-level semantic features and low-level detail features. This approach mitigates the masking of small targets during fusion and improves the detection capability of wildlife targets at different scales in complex environments.

**Figure 7:**
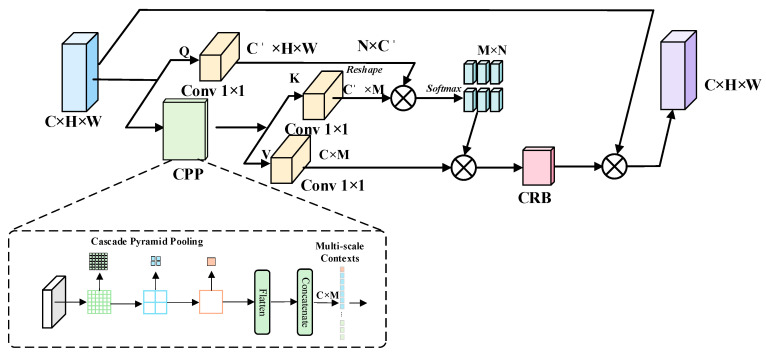
Structure of the CFC module.

The structure of the CFC module is shown in Figure 7. It primarily consists of 1 × 1 convolutions, Cascaded Pyramid Pooling (CPP), and a Context Recalibration Block (CRB), aiming to address semantic discrepancies and background noise during multi-scale feature fusion. Specifically, the input feature map $C\;\times \;\frac{W\;}{2}\;\times \;\frac{H}{2}$ is divided into two branches. The first branch employs a 1 × 1 convolution to generate query features *Q*. The second branch uses the CPP module to construct contextual features *Z* through hierarchical reuse of multi-scale pooling results and further derives key (*K*) and value (*V*) features via convolutional mapping. Subsequently, the correlation between *Q* and *K* is computed, and attention weights *A* are obtained through Softmax normalization. These weights are then applied to aggregate *V*, yielding context-enhanced features. To emphasize effective details and suppress redundant responses, the enhanced features are fed into the CRB for local context recalibration. Finally, the calibrated results are added to the original input *X*, producing the context-calibrated fused features.

**Figure 8:**
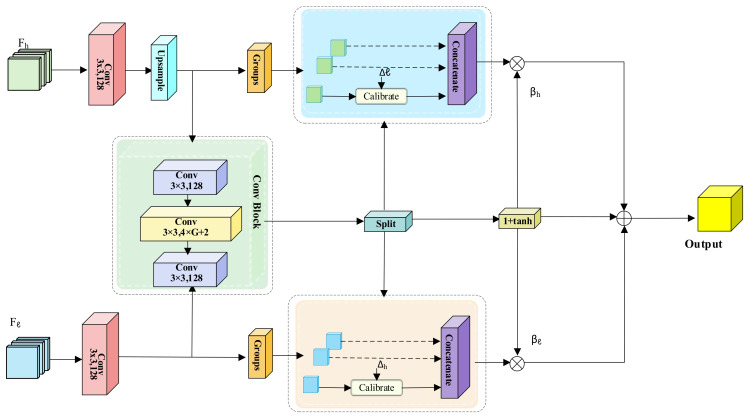
Structure of the SFC module.

The SFC module is primarily designed to address spatial misalignment issues during multi-scale fusion. Its structure is illustrated in Figure 8. Given a low-resolution feature map $F_{h}$ from higher layers and a high-resolution feature map $F_{l}$ from lower layers, the module first adjusts the channel dimensions using 3 × 3 convolutions. Subsequently, $F_{h}$ is upsampled to match the spatial dimensions of $F_{l}$ through bilinear interpolation. This process ensures precise alignment of features for effective fusion. Subsequently, the two feature maps are concatenated along the channel dimension and fed into a Convolution Block. This block predicts two sets of learnable 2D offsets, $\Delta_{h}$ and $\Delta_{l}$, as well as the corresponding dynamic gating factors, $\beta_{h}$ and $\beta_{l}$. This approach facilitates adaptive feature alignment and enhances the fusion process.

During the feature calibration stage, the output of the convolution module undergoes a Split operation, separating the offsets according to high-level and low-level features. The feature maps $F_{h}$ and $F_{l}$ are then divided into *G* groups along the channel dimension. Based on their respective offsets $\Delta_{h}$ and $\Delta_{l}$, bilinear interpolation sampling is applied to each group (Calibrate) to achieve spatial alignment. After sampling, all subgroups are reassembled into the calibrated feature maps $\tilde{F_{h}}$ and $\tilde{F_{l}}$. Finally, a dynamic gating fusion mechanism adaptively controls the contribution ratios of semantic features and detail features in the output, as shown in Equation (7).

(7) $$O=\beta_{h}\cdot \tilde{F_{h}}+\beta_{l}\cdot \;\tilde{F_{l}}$$

Here, $\beta_{h}$ and $\beta_{l}$ are dynamic weight coefficients that regulate the contribution ratios of semantic features and detail features in the fusion results.

In summary, CSFCN leverages the synergistic design of CFC and SFC to achieve contextual semantic calibration and spatial alignment during multi-scale feature fusion. This approach enhances the consistency between high-level semantic features and low-level detail features, providing more discriminative fused features for subsequent detection heads.

#### 2.2.4. Group-Convolution-Based ELGH Lightweight Detection Head

**Figure 9:**
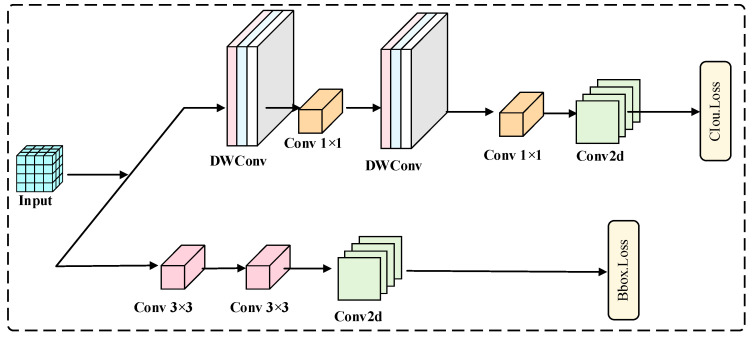
YOLO11 detection head structure.

YOLO11 employs a decoupled structure in the detection head, utilizing separate classification and regression sub-branches for class prediction and bounding box regression, as illustrated in Figure 9. This approach enhances the efficiency and accuracy of the detection process.

The classification branch introduces two depthwise separable convolutions (DWConv) to reduce the number of parameters and enhance computational efficiency. In contrast, the regression branch retains conventional convolution operations to ensure accurate bounding box localization. However, the channel-wise convolution of DWConv weakens cross-channel semantic interactions, adversely affecting texture sensitivity for small objects. Additionally, the computational redundancy resulting from multiple layers of convolution in the original detection head constrains the efficiency of lightweight deployment.

**Figure 10:**
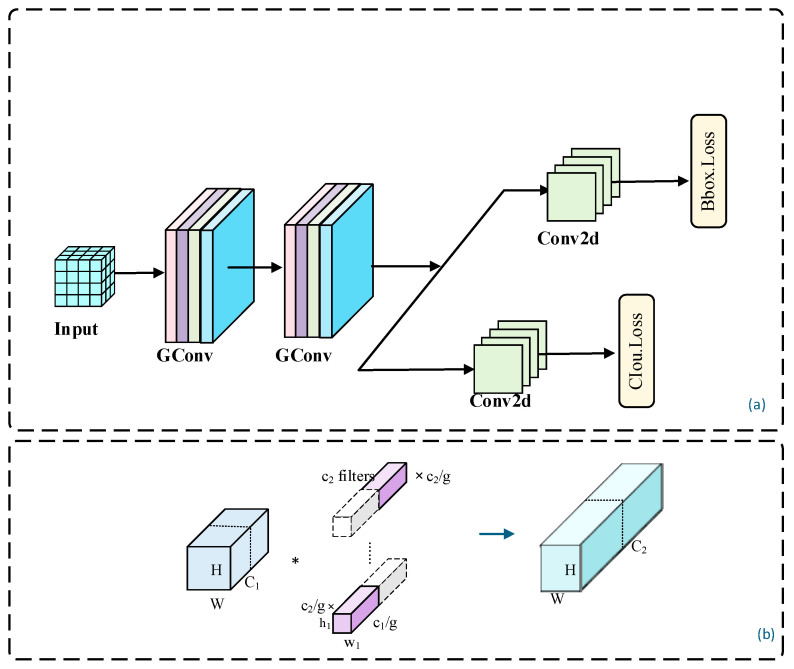
Schematic diagram of the ELGH detection head principle structure. (**a**) Structure of ELGH; (**b**) Structure of GConv. “∗” denotes the multiplication operation.

To address the balance between detection efficiency and accuracy, this study designs an efficient lightweight detection head, ELGH (Efficient Lightweight Group Head), based on group convolution (GConv) [33] as shown in Figure 10a. Structurally, ELGH adopts a decoupled detection head design, with separate branches for classification and regression to perform class prediction and bounding box regression. ELGH replaces the DWConv in the original YOLO11 detection head with two cascaded 3 × 3 group convolutions (GConv), while retaining conventional convolution structures in the regression branch to ensure accurate bounding box localization. Compared to channel-wise convolution of DWConv, GConv maintains a degree of channel interaction within each group, significantly reducing the number of parameters and FLOPs relative to standard convolution. This approach enhances feature modeling efficiency while controlling computational costs, making it more advantageous for object detection in forested environments.

The GConv structure is illustrated in Figure 10b. GConv first partitions the input feature map channels into g groups, with each group containing $c_{1}/g$ channels. The convolution kernels are also divided into g groups, each consisting of $c_{2}/g$ kernels, which convolve only with the corresponding input channel group. The feature maps obtained from each group convolution are concatenated along the channel dimension, resulting in an output feature map with *c*_2_ channels. Accordingly, the parameter amount is reduced from that of a standard convolution, $k_{h}\;\times \;k_{w\;}\times \;c_{1\;}\times \;c_{2},$ to $k_{h}\;\times \;k_{w\;}\times \;g\;\times \;\frac{c_{1}}{g}\;\times \;\frac{c_{2}}{g}$. Therefore, the parameter count and computational load of GConv are approximately *1*/*g* that of standard convolution. By replacing certain standard convolutions with GConv in ELGH, the detection head effectively reduces convolutional computational costs while maintaining intra-group channel interaction and feature modeling capabilities. This approach provides a structural foundation for lightweight deployment in wildlife monitoring scenarios.

## 3. Experiments and Results Analysis

### 3.1. Experimental Environment and Parameter Settings

The experiments were conducted on a Windows 11 (64-bit) operating system. The CPU used was an Intel Core i5-13490F, with 32 GB of RAM. The GPU utilized was an NVIDIA GeForce RTX 4060 Ti, featuring 16 GB of VRAM. The experiments were based on the PyTorch 2.1.1 deep learning framework, with Python version 3.8.18 and CUDA version 12.1. The hyperparameters set for the experiments are presented in Table 2.

### 3.2. Performance Evaluation Metrics

To comprehensively evaluate the performance of the improved model on detection tasks, this study uses Precision (P), Recall (R), mean Average Precision (mAP), Floating Point Operations (FLOPs), and model Parameters (Params) as evaluation metrics.

P represents the proportion of true positives among all samples predicted as positive by the model, as shown in Equation (8):

(8) $$P=\frac{\mathrm{TP}}{\mathrm{TP}+\mathrm{FP}}$$

R represents the proportion of actual positive samples correctly predicted as positive, as shown in Equation (9):

(9) $$R=\frac{\mathrm{TP}}{\mathrm{TP}+\mathrm{FN}}$$

The mAP is used to evaluate a model’s overall detection performance across different classes and confidence thresholds. First, the Average Precision (AP) for a single class is defined as the area under the Precision-Recall curve. Subsequently, mAP is obtained by averaging the AP values across all classes, as shown in Equation (10):

(10) $$\mathrm{mAP}=\frac{1}{m}\sum_{i=0}^{m}\int_{0}^{1}P_{i}\left(R\right)\;\mathrm{dR}$$

This study uses two metrics: mAP@0.5 and mAP@0.5:0.95. The former represents the average precision at an IoU threshold of 0.5. The latter is the mean precision calculated over IoU thresholds from 0.5 to 0.95 with a step size of 0.05.

To assess the model’s deployment potential and computational efficiency, this study focuses on the metrics FLOPs and Params. FLOPs represent the total number of floating-point operations required for a single forward pass. Params refer to the total number of trainable parameters. In general, lower FLOPs and Params are more favorable for deployment on resource-constrained edge devices. However, a balance between detection accuracy and computational efficiency is necessary to achieve an optimal trade-off between performance and deployment cost.

### 3.3. Ablation Study of the Improved Module

To validate the effectiveness of each improved module, this study introduces PMGHA, RFAConv, CSFCN, and ELGH under the same dataset partitioning, training strategy, and evaluation metrics. The detection performance is compared across different combinations of modules. The results are presented in Table 3.

Table 3 shows that the introduction of the four modules improves the baseline model to different degrees, although their contributions mainly focus on different aspects of performance. Specifically, PMGHA, RFAConv, and CSFCN primarily contribute to detection accuracy improvement. The mAP@0.5 values increase to 85.3%, 85.2%, and 85.9%, respectively, while the mAP@0.5:0.95 values rise to 65.2%, 65.1%, and 65.8%. In contrast, ELGH mainly contributes to reducing model complexity. After introducing ELGH, FLOPs decrease to 5.1 G, and the parameter count is reduced to 2.31 M while maintaining relatively stable detection performance. Among the single-module configurations, CSFCN achieves the highest improvement in detection accuracy.

Further analysis of different module combinations shows that combining multiple modules generally leads to better overall performance than single-module configurations. Specifically, after introducing PMGHA and RFAConv, the model achieves Precision, Recall, mAP@0.5, and mAP@0.5:0.95 values of 87.1%, 76.5%, 86.2%, and 65.9%, respectively. When RFAConv and CSFCN are incorporated together, the mAP@0.5 and mAP@0.5:0.95 increase to 86.4% and 66.2%, both exceeding the corresponding single-module results. When PMGHA, RFAConv, and CSFCN are applied simultaneously, the model’s Precision, Recall, mAP@0.5, and mAP@0.5:0.95 further increase to 88.8%, 77.9%, 87.1%, and 67.0%, respectively. These results suggest that the different modules provide complementary effects under the current experimental setting.

When all four modules are integrated, the model achieves the highest overall detection performance under the current experimental setting. Compared with the baseline YOLO11n, the final model improves Precision, Recall, mAP@0.5, and mAP@0.5:0.95 by 4.1%, 2.6%, 3.9%, and 3.4%, respectively. Meanwhile, the model maintains relatively low complexity, with FLOPs and parameter counts of 6.4 G and 2.77 M, respectively. Compared with the three-module configuration, the introduction of the ELGH detection head further reduces computational cost and parameter scale while maintaining detection performance. These results indicate that the proposed lightweight detection head contributes to balancing detection accuracy and model complexity. Overall, the combined integration of the four modules improves detection performance while maintaining relatively low computational complexity.

### 3.4. Random Seed Stability Analysis

To further assess the training stability and reproducibility of the proposed model under different random initializations, the final model, RCAF-Net, was trained five times with only the random seed changed. The dataset split, training strategy, and hyperparameters remained the same. Each run used identical training epochs, optimizer, learning rate, input size, and data augmentation. Test results corresponding to the best validation weights were recorded. The results are shown in Table 4.

As shown in Table 4, the evaluation metrics exhibit relatively small fluctuations under different random seeds. The standard deviations of Precision, Recall, mAP@0.5, and mAP@0.5:0.95 are 0.11, 0.14, 0.11, and 0.12, respectively, indicating relatively stable training performance under different initialization conditions.

**Figure 11:**
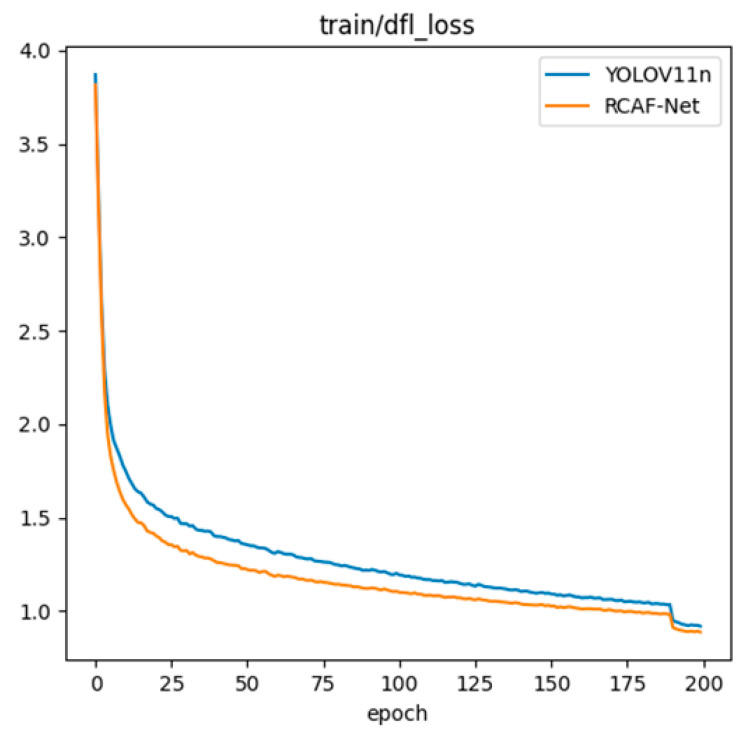
Training loss curves of YOLO11n and RCAF-Net under seed = 0.

To further analyze the training process, the results obtained with seed = 0 were selected as a representative example, and the loss curves of YOLO11n and RCAF-Net are shown in Figure 11. Both models show a gradual decrease in loss and become stable during the later stages of training, indicating an effective optimization process. Compared with YOLO11n, RCAF-Net exhibits relatively lower loss values and smoother fluctuations during most training stages under the current training setting.

### 3.5. Performance Comparison with Mainstream Detection Models

To validate the proposed method’s effectiveness and practicality, RCAF-Net was compared with several mainstream detection models, including Faster R-CNN, RT-DETR [34], and the YOLO series. All models were trained and tested under the same experimental conditions to ensure comparability. Evaluation metrics included Precision (P), Recall (R), mAP@0.5, and mAP@0.5:0.95. Additionally, FLOPs and Params were reported to assess computational cost.

As shown in Table 5, RCAF-Net achieves competitive overall detection performance among the compared models. Compared with Faster R-CNN and RT-DETR, RCAF-Net maintains relatively balanced detection accuracy and model complexity under the current experimental setting. Compared with the baseline YOLO11n, RCAF-Net introduces limited additional complexity while improving Precision, Recall, mAP@0.5, and mAP@0.5:0.95 by 4.1%, 2.6%, 3.9%, and 3.4%, respectively. Among the compared lightweight YOLO models, RCAF-Net achieves relatively higher Precision, mAP@0.5, and mAP@0.5:0.95 values, indicating comparatively balanced detection performance under the current experimental setting.

**Figure 12:**
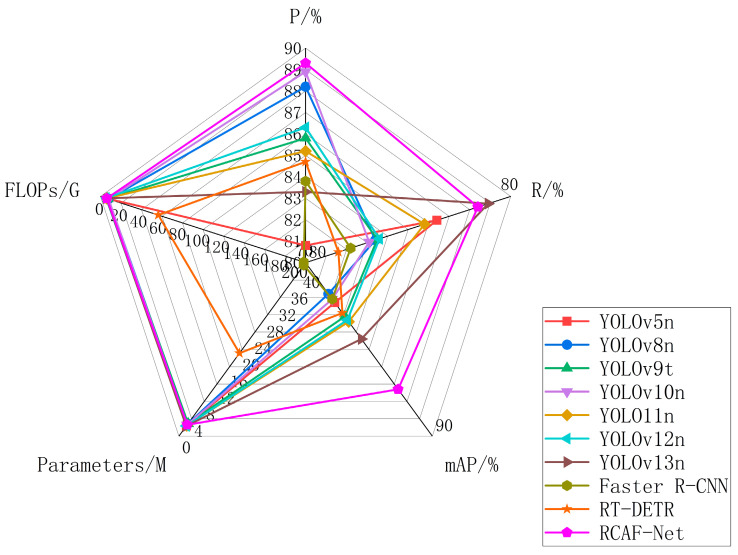
Radar chart of performance comparison among different models.

To provide a clearer visualization of the overall model characteristics, Figure 12 presents a radar chart based on the main evaluation metrics in Table 5. Performance metrics such as Precision, Recall, and mAP@0.5 were positively normalized, where higher values correspond to higher normalized scores. Complexity metrics, including FLOPs and parameter count, were inversely normalized, so lower complexity corresponds to higher normalized scores. After normalization, a larger radar area generally reflects relatively balanced overall performance across the selected metrics. As shown in Figure 12, RCAF-Net exhibits relatively balanced performance in both detection accuracy and model complexity among the compared methods.

### 3.6. Class Recognition Analysis Based on Confusion Matrix

**Figure 13:**
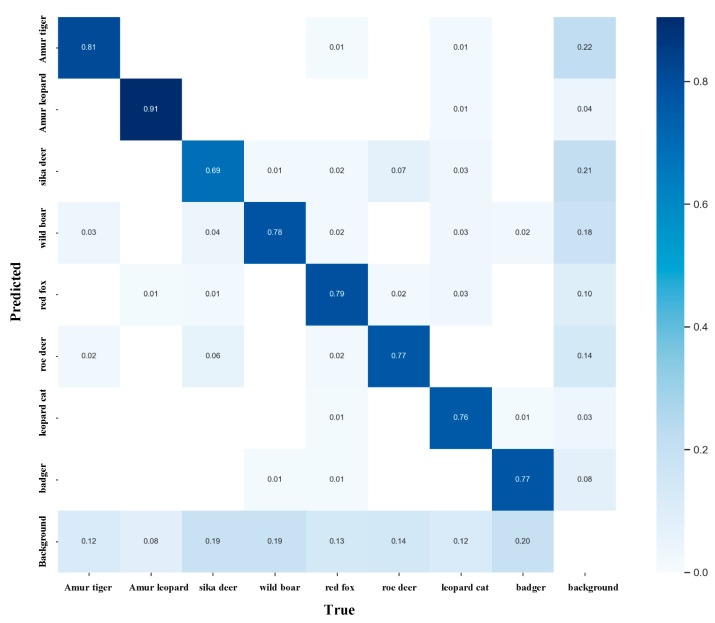
Visualization of the normalized confusion matrix results.

To further analyze the model’s recognition performance across different wildlife categories, a normalized confusion matrix was generated on the test set, as shown in Figure 13.

As shown in Figure 13, relatively high values are concentrated along the main diagonal, indicating that RCAF-Net maintains good discriminative performance for most categories in complex forest scenarios. The diagonal values for the Amur leopard and the Amur tiger are approximately 0.91 and 0.81, respectively, corresponding to relatively higher recognition performance among the evaluated categories. For wild boar, red fox, roe deer, leopard cat, and badger, the diagonal values are approximately 0.78, 0.79, 0.77, 0.76, and 0.77, respectively, indicating relatively stable classification performance. In contrast, the diagonal value for sika deer is approximately 0.69, which is lower than that of the other categories, suggesting relatively greater recognition difficulty.

Further analysis shows noticeable confusion between sika deer and roe deer, with sika deer misclassified as roe deer approximately 0.06 of the time, and roe deer misclassified as sika deer approximately 0.07 of the time. This confusion suggests that distinguishing between the two categories remains challenging under certain monitoring conditions, particularly when target details are limited. In addition, wild boar and badger are misclassified as background at rates of approximately 0.19 and 0.20, respectively, indicating that some small-scale or low-contrast targets remain difficult to distinguish in complex forest scenes. Meanwhile, background regions are misclassified as Amur tiger and sika deer at rates of approximately 0.22 and 0.21, suggesting that certain environmental textures may still interfere with target discrimination in some scenarios.

Overall, although confusion between similar categories and background interference still exists in some cases, the confusion matrix remains mainly concentrated along the diagonal, reflecting relatively consistent category discrimination across most wildlife categories.

### 3.7. Visualization Analysis of Attention Regions Based on Grad-CAM

**Figure 14:**
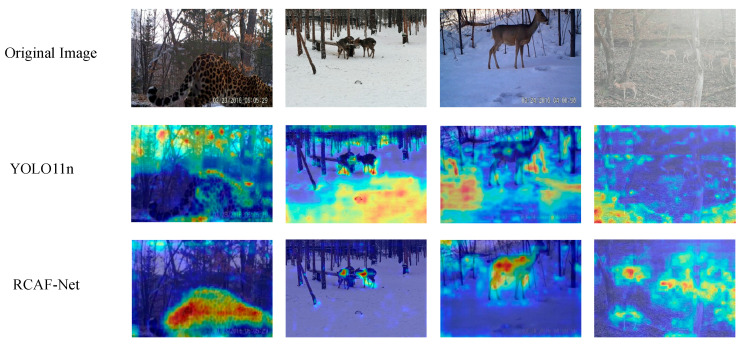
Grad-CAM visualization results for different models. In the heatmap visualization, red indicates higher response intensity, while blue indicates lower response intensity.

To visually analyze the response differences in target regions during the feature extraction stage, Gradient-weighted Class Activation Mapping (Grad-CAM) [35] was applied to visualize the deep feature responses of YOLO11n and RCAF-Net, as shown in Figure 14.

As shown in Figure 14, certain differences can be observed between the Grad-CAM response distributions of YOLO11n and RCAF-Net under different species and scene conditions. In the first column, the response regions of YOLO11n partially extend into surrounding background areas, whereas the responses of RCAF-Net are more concentrated around the Amur leopard target. In the second column, under the snow-covered scene, the high-response regions of YOLO11n are mainly concentrated on the snow-covered background, while the responses of RCAF-Net are more focused on the roe deer target itself. In the third column, the high-response regions of YOLO11n partially extend beyond the roe deer target, whereas the responses of RCAF-Net remain mainly concentrated around the target region. In the fourth column, due to the similarity between the background textures and the sika deer target, YOLO11n does not exhibit an obvious response center, while RCAF-Net maintains relatively more concentrated response regions around the sika deer target. Overall, RCAF-Net shows relatively more concentrated target responses and reduced redundant background responses in some complex scenes.

### 3.8. Comparative Analysis of Detection Results Visualization

**Figure 15:**
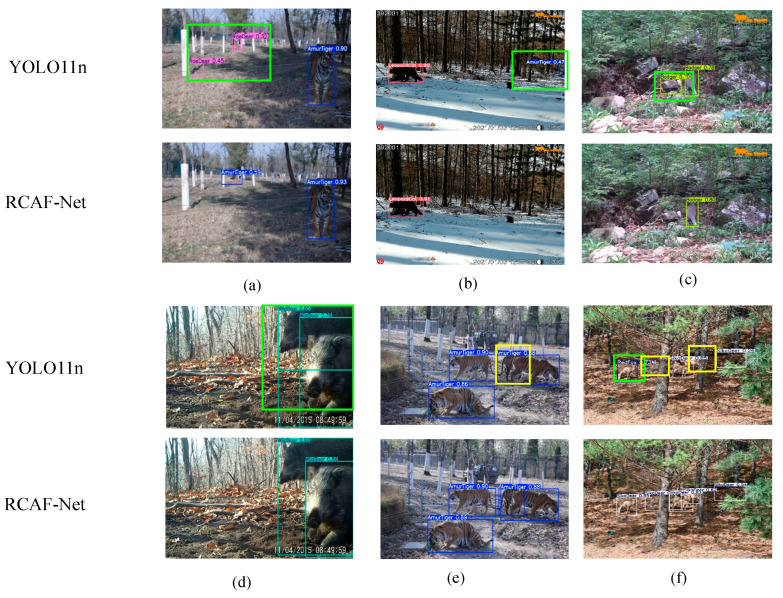
Detection visualization results of different models. (**a**) Amur tiger detection results. (**b**) Leopard cat detection results. (**c**) Badger detection results. (**d**) Wild boar detection results. (**e**) Amur tiger detection results. (**f**) Sika deer detection results. Yellow boxes indicate missed detections, and green boxes indicate false detections.

To further verify whether the observed feature response differences reflect actual detection results, multiple complex scene samples were selected for visualization comparison between YOLO11n and RCAF-Net, as shown in Figure 15.

From the visualization results under complex background conditions, certain differences can be observed between YOLO11n and RCAF-Net across different species scenarios. In Figure 15a, under the multi-scale Amur tiger scene, YOLO11n produces additional detections in surrounding background regions, whereas relatively fewer additional detections are observed in the corresponding RCAF-Net result. In Figure 15b, under the leopard cat scene, YOLO11n additionally detects a distant background target as an Amur tiger, while the corresponding RCAF-Net result shows fewer false detections in the presented scene. In Figure 15c, under the badger scene, YOLO11n incorrectly detects the stone region on the left side as a badger target, whereas fewer false detections are observed in the RCAF-Net result.

The visualization differences are also observable in dense-target and partially occluded scenarios. In Figure 15d, due to the overlap between two wild boar targets, YOLO11n produces additional detections, while the corresponding RCAF-Net result shows relatively clearer separation between adjacent wild boar targets. In Figure 15e, under the overlapping Amur tiger scene, YOLO11n exhibits missed detections for part of the Amur tiger targets, whereas the corresponding RCAF-Net result preserves more Amur tiger detections in the presented example. In Figure 15f, under the sika deer scene, YOLO11n exhibits both missed detections and false detections under conditions involving occlusion, overlapping targets, tree shadow interference, and multi-scale target distribution, while relatively fewer incorrect detections are observed in the RCAF-Net result.

Overall, these visualization results are consistent with the quantitative evaluation to some extent and provide qualitative evidence of the model’s detection behavior under complex monitoring conditions.

### 3.9. Cross-Dataset Generalization Performance Analysis

**Figure 16:**
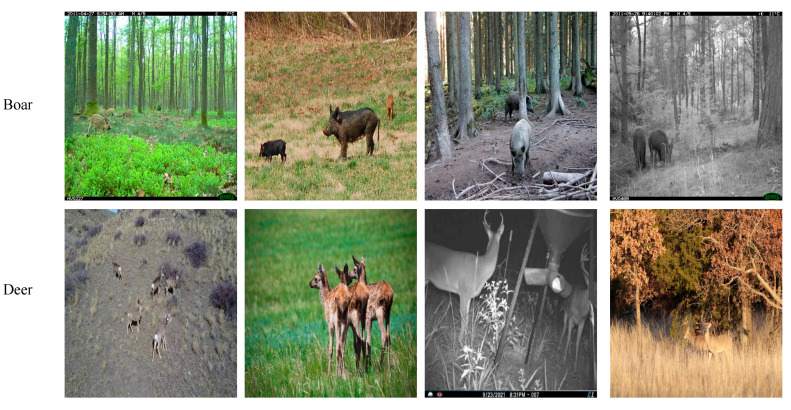
Sample images from the dataset.

To further validate the generalization ability of the model, this study selected the wildlife Computer Vision Model dataset published on the Roboflow Universe platform for cross-dataset testing [36]. This dataset contains two target classes: deer and wild boar, totaling 1637 annotated images. The dataset is divided into training, validation, and test sets, consisting of 1309, 165, and 163 images, respectively (some examples are shown in Figure 16). This dataset is completely independent of the custom dataset developed in this study. It effectively evaluates the detection performance and robustness of the RCAF-Net model on external public data.

The experimental results are presented in Table 6. The proposed model demonstrates strong detection performance on the public wildlife Computer Vision Model dataset. Precision, mAP@0.5, and mAP@0.5:0.95 reach 88.22%, 87.09%, and 47.46%, respectively, all exceeding those of the baseline model YOLO11n. Notably, mAP@0.5 shows an improvement of 5.34%, indicating that the proposed model effectively enhances target detection accuracy in external data environments. Additionally, mAP@0.5:0.95 improves by 2.78%, further confirming that the model maintains better prediction box quality and localization ability under stricter evaluation criteria. Overall, although Recall shows a slight decrease, the improvements in precision and overall detection performance are more pronounced. This reflects the effectiveness and generalization potential of the proposed enhancement strategy in cross-dataset testing.

**Figure 17:**
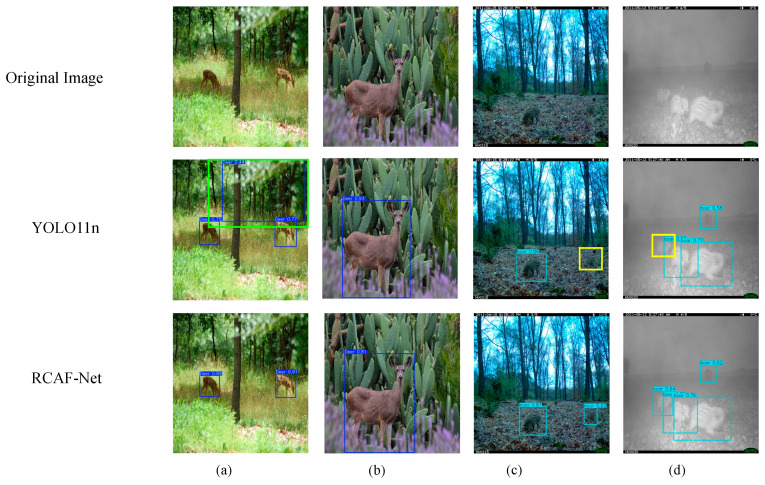
Visualization of detection results. (**a**) Deer detection results. (**b**) Deer detection results. (**c**) Wild boar detection results. (**d**) Wild boar detection results. Yellow boxes indicate missed detections, while green boxes indicate false detections.

To further analyze the specific detection performance of the model, four representative samples were selected for visual comparison, as shown in Figure 17. In Figure 17a, under a forest-grass background, branches and trunks are morphologically similar to local structures such as deer legs, and the background textures are intertwined with the target boundaries, causing YOLO11n to falsely detect non-target regions as deer, whereas RCAF-Net effectively suppresses such background interference. In Figure 17b, both models are able to detect the deer target, but RCAF-Net yields higher prediction confidence. In Figure 17c,d, YOLO11n shows missed detections of wild boars in both cases. Specifically, in Figure 17c, the wild boar closely resembles the fallen leaves and the textures of surrounding branches. In Figure 17d, the wild boar is partially occluded under low-contrast conditions, resulting in insufficient target feature representation and increasing the difficulty of detection.

### 3.10. Embedded Edge Device Deployment Validation

**Figure 18:**
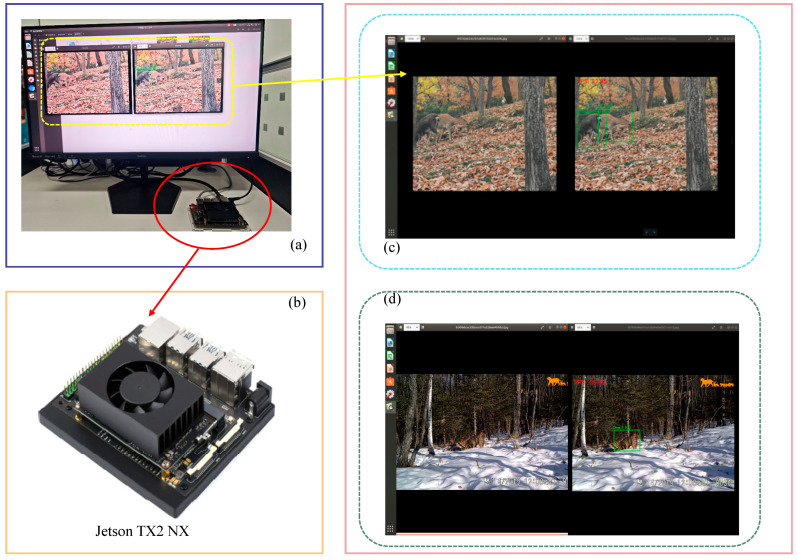
Deployment of RCAF-Net on an embedded edge device. (**a**) Deployment test scene; (**b**) Jetson TX2 NX development board; (**c**,**d**) detection results under different monitoring scenarios.

To verify the practical application potential of the proposed model for wildlife monitoring in forested areas and to further assess its deployment feasibility on resource-constrained edge devices, the Jetson TX2 NX was selected as the embedded deployment platform. Edge testing was conducted on the trained model, with the deployment process and results illustrated in Figure 18. Panel (a) shows the actual deployment test scenario. Panel (b) presents a physical image of the Jetson TX2 NX development board. Panels (c) and (d) provide examples of the model’s detection results across different wildlife monitoring scenarios.

The software environment for this deployment experiment was Ubuntu 20.04.5 LTS, configured with Python 3.8 and PyTorch 1.8.0. The hardware utilized was the Jetson TX2 NX, which features a dual-core NVIDIA Denver2 processor, a quad-core ARM Cortex-A57 CPU, and a 256-core NVIDIA Pascal GPU. It operates at a power consumption of only 7.5 W, providing edge inference support for the object detection model. In northeastern forest areas of China, wildlife monitoring typically requires infrared cameras, patrol terminals, or front-end intelligent devices. These tools are essential for continuous sensing and on-device inference. Therefore, runtime efficiency and continuous inference capability are important considerations for practical applications.

To further improve inference efficiency on edge devices, the trained RCAF-Net model was additionally optimized using TensorRT FP16 inference acceleration. The PyTorch model was first exported to ONNX format, and an FP16 inference engine was then constructed using TensorRT 8.4. During deployment, FP16 precision inference was enabled through the TensorRT runtime environment. After FP16 optimization, the inference speed of the model on the Jetson TX2 NX platform increased from approximately 18 FPS to 27 FPS, representing an improvement of approximately 50% in inference speed. Meanwhile, the decrease in detection accuracy remained below 0.5% in mAP@0.5, indicating that the optimized model maintained relatively stable detection performance while improving inference efficiency.

The experimental results indicate that the deployed model is capable of continuously processing video streams on the Jetson TX2 NX platform, meeting the basic requirements for real-time or near-real-time wildlife monitoring tasks. Figure 18c,d present representative deployment results under different monitoring conditions. Additionally, the model’s inference process remained smooth during operation, with no obvious stuttering, interruptions, or prolonged delays observed during continuous inference. This further suggests the potential applicability of RCAF-Net for edge-assisted wildlife monitoring tasks under the current experimental setting.

## 4. Discussion

RCAF-Net, developed in this study, showed good detection stability under complex backgrounds, occlusion interference, and small distant target scenarios. Comparative experiments, confusion matrix analysis, heatmaps, and detection visualizations consistently indicated that the improved model could focus more effectively on target regions in complex natural scenes and maintain robust detection performance under multi-target coexistence and partial occlusion. Compared with the baseline model, these improvements were reflected not only in the overall detection results but also in the model’s stronger ability to distinguish valid targets from non-target regions in cluttered backgrounds, suggesting that task-oriented optimization is necessary for wildlife monitoring in the forest region of Northeast China.

From a monitoring perspective, these findings are of practical relevance. Field camera monitoring usually produces a large volume of raw images, while complex backgrounds, illumination changes, and target scale variation further increase the difficulty of manual screening. Under such conditions, the more stable detection performance of RCAF-Net can improve the efficiency of automated image pre-screening and reduce the influence of obvious false positives and false negatives on subsequent data processing, thereby providing more reliable methodological support for species occurrence information extraction and the use of monitoring data.

Several limitations should also be acknowledged. First, confusion remains among some categories, indicating that the model still has room for improvement in representing fine-grained differences under conditions involving visually similar species, partial visibility, or low contrast. Second, the present study was mainly based on static images, and temporal information from continuous video was not exploited. Third, the current dataset covers typical scenarios such as forests, snow-covered areas, and shrubs. However, its adaptability to nighttime infrared imagery and extreme weather conditions still needs validation. Additionally, data from broader geographic regions requires further assessment. Therefore, future work may focus on expanding multi-region, multi-season, and multimodal data. It will also address temporal modeling and long-term edge deployment evaluation. These efforts aim to enhance the model’s robustness and applicability under real-world wildlife monitoring conditions.

## 5. Conclusions

This study addressed several challenges in wildlife detection in complex natural environments, including complex background interference, frequent occlusions, and difficulties in detecting distant, small targets with significant scale variations. Based on YOLO11n, an improved wildlife-target detection model, RCAF-Net, was proposed for complex forest monitoring scenarios in northeastern China. By integrating PMGHA, RFAConv, CSFCN, and ELGH, the proposed method enhanced feature representation, multi-scale feature fusion, and lightweight deployment capability under complex natural scenes. Experimental results demonstrated that RCAF-Net achieved improved detection performance and deployment efficiency under challenging forest monitoring conditions, with mAP@0.5 improving by 3.9% over YOLO11n while maintaining relatively low computational complexity. Deployment experiments on the Jetson TX2 NX platform showed that the proposed model achieved approximately 27 FPS, indicating its potential applicability for edge-assisted wildlife monitoring.

In conclusion, RCAF-Net improves wildlife detection accuracy in complex forest scenes. Its potential for real-world monitoring workflows was validated via embedded deployment, offering a viable technical path for automated monitoring. However, limitations remain, including dataset scale, species diversity, environmental variations, and potential class imbalance. Future work will expand dataset diversity and scale, while optimizing model accuracy and lightweight design to enhance robustness and real-time performance in practical applications.

## Figures and Tables

**Table 1:** Animal data distribution statistics.

Class	Train	Valid	Test
Amur Tiger	439	125	64
Amur Leopard	313	89	46
Sika Deer	398	113	58
Wild Boar	444	127	64
Red Fox	352	100	51
Roe Deer	443	126	64
Leopard Cat	215	61	32
Badger	261	74	39
Total	2865	815	418

**Table 2:** Training parameter value settings.

Parameter	Setting
Epochs	200
Patience	50
Batch size	8
Images size	640
Workers	8
Optimizer	SGD
Close mosaic	10
Warmup epochs	3
Initial Learning Rate	0.01
Final Learning Rate	0.01
Momentum	0.937
Weight decay	0.0005

**Table 3:** Ablation study results.

Model	P/%	R/%	mAP@0.5/%	mAP@0.5:0.95/%	FLOPs/G	Params/M
YOLO11n	85.2	75.8	83.4	63.9	6.3	2.58
PMGHA	86.7	76.4	85.3	65.2	6.5	2.59
RFAConv	86.3	76.2	85.2	65.1	6.9	2.69
CSFCN	87.4	75.9	85.9	65.8	7.2	2.96
ELGH	86.2	75.9	85.2	65.1	5.1	2.31
PMGHA + RFAConv	87.1	76.5	86.2	65.9	6.6	2.61
RFAConv + CSFCN	87.9	76.8	86.4	66.2	7.3	2.98
PMGHA + RFAConv + CSFCN	88.8	77.9	87.1	67.0	7.6	2.98
PMGHA + RFAConv + CSFCN + ELGH	89.3	78.4	87.3	67.3	6.4	2.77

**Table 4:** Analysis of improved model stability.

Model	Seed	P/%	R/%	mAP@0.5/%	mAP@0.5:0.95/%
RCAF-Net	0	89.3	78.4	87.3	67.3
RCAF-Net	42	89.1	78.2	87.1	67.1
RCAF-Net	123	89.4	78.5	87.4	67.4
RCAF-Net	2024	89.2	78.3	87.2	67.2
RCAF-Net	999	89.3	78.6	87.3	67.4
RCAF-Net	Mean	89.26	78.40	87.26	67.28
RCAF-Net	Std	0.11	0.14	0.11	0.12

**Table 5:** Performance comparison of different mainstream detection models.

Model	P/%	R/%	mAP@0.5/%	mAP@0.5:0.95/%	FLOPs/G	Params/M
YOLOv5n	80.8	76.4	82.3	62.8	7.1	2.50
YOLOv8n	88.2	73.4	81.8	63.3	8.1	3.00
YOLOv9t	85.8	73.5	83.1	63.6	7.4	3.05
YOLOv10n	88.9	73.1	82.1	63.2	8.2	2.70
YOLO11n	85.2	75.8	83.4	63.9	6.3	2.58
YOLOv12n	86.3	73.6	83.3	65.9	5.8	2.50
YOLOv13n	83.3	78.9	84.4	65	6.1	2.45
Faster R-CNN	83.8	72.2	82.1	61.8	208.1	41.40
RT-DETR	84.7	71.6	82.9	64.1	59.2	20.10
RCAF-Net	89.3	78.4	87.3	67.3	6.4	2.77

**Table 6:** Comparison of cross-dataset generalization performance between YOLO11n and RCAF-Net on the public dataset.

Model	P/%	R/%	mAP@0.5/%	mAP@0.5:0.95/%
YOLO11n	86.25	79.84	81.75	44.68
RCAF-Net	88.22	79.03	87.09	47.46

## Data Availability

The original contributions presented in the research are included in the article. Further inquiries can be directed to the corresponding author.
